# Advances in modelling the risk of benign and malignant lung nodules

**DOI:** 10.3389/fonc.2025.1648548

**Published:** 2025-10-23

**Authors:** Shang Du, Tangwei Wu, Hui Wang, Zheqiong Tan, Zhongxin Lu

**Affiliations:** ^1^ School of Laboratory Medicine, Hubei University of Chinese Medicine, Wuhan, China; ^2^ Department of Medical Laboratory, The Central Hospital of Wuhan, Tongji Medical College, Huazhong University of Science and Technology, Wuhan, China

**Keywords:** lung nodule, predictive model, biomarker, lung cancer, application

## Abstract

Lung nodules are critical indicators for early lung cancer detection, yet accurately distinguishing between benign and malignant lesions remains a clinical challenge. This review summarizes advances in predictive models for lung nodule risk assessment, spanning classical clinical-imaging models, biomarker-based approaches, and artificial intelligence (AI)-driven tools. While classical models provide a foundational framework, their performance often varies across populations. Biomarkers and AI models significantly enhance diagnostic precision by capturing molecular and imaging features imperceptible to the human eye. However, issues such as generalizability, standardization, and data security persist. The most promising direction lies in multimodal integration, combining clinical, imaging, biomarker, and AI data to achieve superior accuracy with an area under the curve (AUC) >0.90. Future efforts should focus on multi-center validation, standardized biomarker assays, and data secure, scalable AI systems to translate these innovations into routine clinical practice, enabling personalized and early lung cancer diagnosis.

## 1 Introduction

Lung cancer is the most frequently diagnosed cancer worldwide and is one of the leading cause of cancer-related mortality (1). Lung nodules are defined as round or oval lesions (≤3 cm in diameter) within the lungs, detectable through imaging, and serve as critical indicators of early-stage lung cancer. These nodules are typically identified using CT scan or chest X-ray (2). Statistically, the detection rate of nodules via chest X-ray is 2.1%, whereas CT scans can achieve a detection rate of up to 17.0%, primarily due to the superior spatial resolution offered by CT technology (3). The widespread application of CT technology has led to a marked increase in the detection rate of lung nodules (4). Furthermore, the integration of advanced imaging techniques, such as high-resolution CT combined with Positron Emission Tomography (PET), has significantly enhanced the differential diagnosis of lung nodules, facilitating early lung cancer screening (5). The characteristics of nodules, including size, number, and location, are significantly correlated to the risk of lung cancer (6, 7). Nonetheless, due to the potential for false positives in imaging screenings, the definitive determination of nodule characteristics should be corroborated with other examinations (8). It is imperative to develop a comprehensive diagnostic framework in clinical practice that integrates the imaging attributes of lung nodules, pathological assessments, laboratory examinations and individual patient risk factors.

Tumor markers serve as valuable adjuncts in the diagnosis and management of lung cancer. Although lacking sufficient sensitivity or specificity for primary screening, they provide critical information that complements imaging findings. Commonly utilized serum markers include Carcinoembryonic Antigen (CEA), Squamous Cell Carcinoma Antigen (SCCA), Cytokeratin-19 fragments (CYFRA 21-1), and Neuron-Specific Enolase (NSE). In addition, novel biomarkers including microRNAs (miRNAs), autoantibodies, circulating tumor cells (CTCs), and circulating abnormal cells (CACs) have been developed in recent years for the diagnosis of lung cancer, which holds significant promise for improving personalized diagnosis of lung cancer (9). However, challenges remain in the clinical application of these biomarkers, including the need for standardization of detection methods, variable specificity, and high costs for certain markers.

As the development of AI, deep learning, random forest algorithms, and ensemble learning methods have revolutionized the discrimination of benign and malignant pulmonary nodules on CT scans (10, 11). AI models automatically learn to extract complex radiographic features—such as size, shape, texture, and growth patterns—that are often imperceptible to the human eye, providing a quantitative assessment of malignancy risk. However, performance can also be hampered by limited generalizability if models are trained on non-diverse datasets from specific scanners or populations.

Despite these current limitations of each kind of prediction models, their integration into multimode represents the most promising direction for enhancing diagnostic accuracy of lung nodules (12). However, a comprehensive and up-to-date synthesis that systematically compares classical models with emerging biomarker-based and AI-driven approaches, while critically evaluating their integration and translational challenges, is lacking. Taken together, this review aimed to summarize the characteristics of malignancy risk predictive models for lung nodules, underscoring the advantages and limitations of each category of models, based on previous studies searching from PubMed using the key words as lung nodules, predictive model, biomarkers, AI or multimode in recent ten years. Moreover, we mainly focused on the emerging biomarkers, AI-based technology and methodological innovations in this area, and offered insights into the potential clinical applications of these models. In addition, we discussed about the comparative performance of models across diverse populations and the prospective directions of data security as the application of large cohort and AI tools. By providing a holistic overview, this review fills a critical gap in the literature and serves as a valuable resource for clinicians and researchers navigating the rapidly evolving landscape of lung nodule risk stratification. We also highlight the pressing issues that warrant further investigation, such as the need for standardized biomarker assays, multi-center validation of AI algorithms to ensure generalizability, and the development of secure data-sharing frameworks for model training.

## 2 Classical predictive model

### 2.1 Mayo model

The Mayo model represents one of the pioneering predictive frameworks for assessing the probability of malignancy in pulmonary nodules and has been extensively utilized in clinical settings (13). This model was formulated using clinical data from 629 patients with isolated pulmonary nodules who were evaluated at the Mayo Clinic in the United States between 1984 and 1986. The cohort was divided into a development group comprising 419 patients and a validation group consisting of 210 patients. The overall malignancy rate among the nodules was 23%, with diameters ranging from 4 to 30 mm. Benign nodules were identified through imaging studies that demonstrated stable, shrinking, or disappearing nodule sizes over a period of at least two years, while malignant nodules were confirmed via pathological diagnosis. The equation for the Mayo model is as follows: the probability of malignancy P = e^x^/(1 + e^x^), where x = -6.8272 + [0.0391 × age (years)] + (0.7917 × history of smoking) + (1.3388 × history of malignant tumor) + [0.1274 × diameter (mm)] + (1.0407 × spiculation) + (0.7838 × upper lobe). The area under the curve (AUC) for the model’s receiver operating characteristic (ROC) curve was 0.8328 ± 0.0226 in the development group and 0.8014 ± 0.0360 in the validation group, indicating robust predictive performance.

The Mayo model offers the advantage of utilizing clinical and imaging data that are both readily accessible and straightforward to implement in clinical practice. Nonetheless, the model presents several limitations. Firstly, it was developed over three decades ago, and its relevance may be compromised due to evolving disease patterns and advancements in imaging technology. Secondly, the model primarily depends on CT imaging features, which may restrict its predictive capability for certain complex or atypical nodules. Additionally, research has indicated that the Mayo model exhibits suboptimal predictive performance in the Chinese population, evidenced by an AUC value of only 0.653, thereby suggesting its limited applicability across diverse populations (14). Overall, the Mayo model has a certain degree of accuracy in predicting the degree of malignancy of lung nodules, but with the development of medical technology, its limitations have gradually appeared, and it may be necessary to combine with other more advanced models or techniques to improve the accuracy of its prediction in clinical practice.

### 2.2 VA model

The VA model utilized data pertaining to lung nodules sourced from the United States Department of Veterans Affairs. This study included 375 patients, with a lung cancer diagnosis rate of 54% (15). The inclusion criteria specified that patients must have a newly identified solitary lung nodule measuring between 7 and 30 mm in diameter as observed on X-ray. Nodules were classified as benign based on pathological findings indicative of benignity or the nodule size remained stable over a two-year period. Conversely, nodules were deemed malignant if the pathological diagnosis suggested malignancy.

The VA model employed a cross-validation approach to enhance the model’s accuracy and reliability. Specifically, the researchers implemented a 10-fold cross-validation method, whereby the study population was randomly divided into 10 equal subsets. The model was trained using data from 9 subsets and tested on the remaining subset, with this process repeated 10 times, each time with a different test subset. Independent predictors integrated into the model included smoking duration, age, maximum nodule diameter, and time since smoking cessation. The VA model equation was P=e^x^/(1+e^x^), x=-8.404+(2.061×smoking history)+(0.779×age/10)+(0.112×diameter)-(0.567×time to quit smoking/10). The AUC of the ROC model was 0.79, which indicating its accuracy in predicting the degree of malignancy of lung nodules. The calibration curve demonstrated that the predicted probabilities were in strong concordance with the observed probabilities.

The model showed good discrimination and calibration with stable internal validation and high malignant prevalence. However, the study only focused on elderly male veterans and lacked women, younger participants and external validation, limiting its generalizability. In addition, the measuring of lung nodules from X-ray was less precise than the CT imaging features, which also weakened the explanatory power of this model.

### 2.3 Brock model

The Brock model, alternatively referred to as the Pan Can model or McWilliams model (16). This study aimed to identify factors that could predict whether a lung nodule detected during initial low-dose CT screening was malignant or would be diagnosed as malignant upon follow-up examination. The training dataset was sourced from the Pan-Canadian Early Detection of Lung Cancer Study (Pan Can), while the validation dataset was obtained from the British Columbia Cancer Agency (BCCA). The training dataset comprised 1,871 participants with a total of 7,008 lung nodules, whereas the validation dataset included 1,090 participants with 5,021 lung nodules. The cancer incidence rates were 5.5% for the training set and 3.7% for the validation set, reflecting a low incidence of malignant nodules in both cohorts. The study excluded individuals with no history of smoking, a history of previous tumors, and those younger than 50 years or older than 75 years. The lung nodules examined in the study ranged from 1 to 86 mm in diameter.

The analytical approach employed was multifactorial logistic regression analysis, incorporating variables identified as lung cancer risk factors in the literature as well as those routinely associated with the disease. The diagnostic criteria were based on the pathological findings of the patients, thereby minimizing bias from unknown or incorrect diagnoses and ensuring a high degree of reliability in the results. Ultimately, the variables included in the model included gender, diameter, spiculation, and location. Brock’s model equation: p=ex/(1+ex), x=-6.6144+(0.6467×gender)-[5.5537×diameter in millimeters]+(0.9309×spiculation)+(0.6009×superior lobe). This model integrates multiple imaging features and clinical data to provide a comprehensive assessment. The Brock model demonstrates high predictive accuracy; for instance, in a comparative study, the AUC of the Brock model was 0.902, surpassing the AUC of the Mayo model, which was 0.895, thereby indicating the superior predictive accuracy of the Brock model (17).

The model was developed based on two prospective, multicenter, population-based lung cancer screening cohorts, resulting in good generalizability. Moreover, it included an online calculator. It uses the Brock model to predict the likelihood of lung nodule malignancy. By entering basic patient and nodule details, it provides an immediate malignancy estimate. It is scientifically rigorous, highly accurate, free, and easy to use, aiding clinicians in making informed, low-risk decisions regarding lung nodules. The model also uniquely confirmed that no malignant risk for peri segmental nodules, offering valuable clinical insights. However, this model only focused on high-risk smokers, making it less applicable to broader groups.

### 2.4 PKUPH model

The Peking University People’s Hospital (PKUPH) model represents the inaugural predictive framework for assessing the probability of malignancy in pulmonary nodules, developed within China utilizing domestic patient data. This model was formulated through multifactorial logistic regression analysis using the clinical data from patients who underwent surgical intervention and received a pathological diagnosis of Solitary Pulmonary Nodule (SPN) at PKUPH between 2000 and 2009 (18). 371 patients were included in the development cohort and 62 patients were included in the validation cohort. The model integrates variables including patient age, maximum nodule diameter, family history of tumors, calcification, spiculation sign, and nodule borders. The model’s performance is characterized by an AUC value of 0.754, a sensitivity of 69.51%, and a specificity of 73.55%. The prediction formula of the PKUPH model was P=ex/(1+ex), and x=-4.496+(0.070×age) + (0.676×maximum tumor diameter) + (0.736 × spiculation sign) + (1.267 × family history of tumor) - (1.615 × calcification) - (1.408 × well defined border).

In a comparative study of the PKUPH and Mayo models, the PKUPH model demonstrated superior discriminatory capability in differentiating between malignant and benign nodules (19). Notably, nodule calcification was identified as an independent risk factor in this model, which is not emphasized in other international models and deserves more attention in future studies. The PKUPH model is particularly pertinent to the Chinese population limiting its application. Additionally, variable assessment depends on subjective physician descriptions, affecting its reproducibility and generalizability, which require further validation. The key characteristics of the classical predictive models for lung cancer are listed (**Table 1**).

**Table 1:** key characteristics of classical diagnostic models for lung cancer.

Authors (year)	Study design	Population	Data source	Predictors used	Calibration	AUC* (95%Cl)	Sensitivity	Specificity	Reference
Swensen et al. (1997)	Retrospective cohort study	American (1984.1.1-1986.5.1) n=419(Training set) n=210(Validation set)	Single center	Age, smoking history, prior history of cancer, diameter, spiculation, upper lobe location	Excellent	0.8014	93%	47%	(13)
Gould et al. (2007)	Retrospective cohort study	American (1988.9-2001.6) n=375(Training set) No independent validation set	Multicenter	Age, smoking history, Time since quitting smoking, nodule size	Excellent	0.79 (0.74–0.84)	Not reported	Not reported	(15)
McWilliams et al. (2013)	Prospective study	Canada (2008.9-2010.12) n=1871(Training set) n=1090(Validation set)	Multicenter	Gender, diameter, spiculation, superior lobe	Excellent	0.938 (0.872–0.978)	71.4%	95.5%	(16)
Li et al. (2011)	Retrospective cohort study	China (2000.1-2010.5) n=371(Training set) n=62(Validation set)	Single center	Age, diameter, spiculation, family history of tumor, calcification, clear border	Not calibrated	0.888	92.5%	81.8%	(18)

AUC, area under curve.

## 3 Biomarker-based predictive models

Biomarkers are observable and quantifiable indicators reflecting physiological or pathological states of human body, which facilitates the diagnosis, prediction, and treatment of diseases (20). Classic tumor markers like CEA, CYFRA 21-1, and NSE assist in diagnosing lung cancer due to their non-invasive, allowing for treatment monitoring and early detection of recurrence. However, they have notable drawbacks as low sensitivity and specificity. Thus, they are not definitive diagnostic or screening tools and should be used alongside imaging and pathology for a complete clinical evaluation (21). In recent years, the development of predictive models based on biomarkers has emerged as a significant research focus, particularly in differentiating benign from malignant lung nodules, which is crucial for the early diagnosis of lung cancer (22). The integration of multiple biomarkers, such as circulating tumor RNA (ctRNA), circulating tumor DNA (ctDNA), DNA methylation, and lung cancer-related proteins, with clinical data and imaging characteristics enhances diagnostic precision while minimizing the need for invasive procedures. Here compares the main features of previously published biomarker-based lung-cancer predictive models (**Table 2**).

**Table 2:** key characteristics of Biomarker-based diagnostic models for lung cancer.

Authors (year)	Study design	Population	Data source	Predictors used	Calibration	AUC* (95%Cl)	Sensitivity	Specificity	Reference
Li et al. (2020)	Retrospective cohort study	China (2000.1-2020.4) No specific quantity has been explicitly stated	Multicenter	CEA*	Not calibrated	0.77(0.73-0.80)	33%	92%	(23)
Hou et al. (2024)	Retrospective cohort study	China (2016.5-2021.5) n=239(Training set) n=103(Validation set)	Single center	CEA*, CYFRA21-1*, Radiomics Score	Excellent	0.76(0.66-0.86)	84.3%	63.6%	(24)
Zhang et al. (2024)	Multi-stage validation study	China (2017.11-2020.1) n=168(Training set) No independent validation set	Multicenter	PRDX2*, PON1*, APOC3*, lobulation, spiculation, vascular, indentation, CEA*, CA125*, CYFRA21-1*	Not calibrated	0.904(0.859-0.949)	81.4%	90.1%	(25)
Ostrin et al. (2021)	Retrospective case-control study	American (2004–2019) n=200(Training set) n=60(Validation set)	Multicenter	pro-SFTPB*, CEA*, CYFRA21-1*, CA125*	Not calibrated	0.90	99.6%	28%	(26)
Kuang et al. (2019)	Retrospective cohort study	China n=63(Training set) No independent validation set	Single center	FGB*, FGG*	Not calibrated	0.794(0.681-0.908)	81.4%	70.0%	(28)
Guida et al. (2018)	Retrospective cohort study	Europe n=324(Training set) n=153(Validation set)	Multicenter	CEA, CA125, CYFRA21-1, Pro-SFTPB*, smoking	Not calibrated	0.83 (0.76–0.90)	63%	83%	(30)
Zhou et al. (2019)	Prospective cohort study	China (2013.10-2016.9) n=260(Training set) n=122(Validation set)	Multicenter	FR+-CTC*, CEA*, CYFRA21-1*, NSE*	Excellent	0.802(0.673-0.930)	55.8%	81.3%	(32)
Ren et al. (2025)	Prospective cohort study	China (2018–2023) n=76(Training set) No specific quantity has been explicitly stated	Single center	CTC count + uAI* platform risk stratification	Excellent	0.805	Not reported	Not reported	(33)
Yang et al.(2022)	Retrospective diagnostic study	China (2020.5-2021.4) n=93(Training set) No independent validation set	Single center	PNAIDS*, CAC*	Not calibrated	0.847 (0.769–0.924)	61.0%	94.1%	(48)
Tahvilian et al. (2023)	Prospective cohort study	American (2018.12-2021.2) n=151(Training set) No independent validation set	Multicenter	CAC*	Excellent	0.78(0.70–0.87)	77%	72%	(49)
Li et al. (2017)	Prospective observational pilot study	China (2015.3-2015.11) n=39(Training set) No independent validation set	Single center	Age, smoking history, emphysema, nodule diameter, spiculation, vascular sign, CYFRA21-1*, CEA*, miRNA-21-5p, miRNA-574-5p	Not calibrated	0.921	Not reported	Not reported	(53)
Lin et al. (2017)	Prospective cohort study	North America and China n=135(Training set) n=224(Validation set)	Multicenter	miR-205-5p, miR-126-3p, Maximum diameter of the nodule	Not calibrated	0.94	88.9%	90.5%	(54)
Yu et al. (2017)	Retrospective cohort study	China (2012–2013) n=80(Training set) No specific quantity has been explicitly stated	Single center	miR-92a-2	Not calibrated	0.761 (0.658–0.864)	56%	100%	(56)
Yuan et al. (2020)	Prospective observational diagnostic study	China (2015.11-2017.12) n=265(Training set) n=223(Validation set)	Single center	RMRP*, NEAT1*, TUG1*, MALAT1*	Not calibrated	0.89(0.84–0.94)	86.96%	74.65%	(64)
Xie et al. (2018)	Retrospective cohort study	China (2010–2012) n=260(Training set) n=200(Validation set)	Single center	SOX2OT*, ANRIL*, CEA*, CYFRA21-1*, SCCA*	Not calibrated	0.883 (0.830–0.924)	91.0%	70.0%	(65)
Lamiaa M Kamel et al., 2019	Retrospective cohort study	American (2018–2021) n=485(Training set) n=261(Validation set)	Single center	GAS5*, SOX2OT*	Not calibrated	0.90	83.8%	81.4%	(66)
Jiang et al. (2018)	Retrospective cohort study	China (2014–2017) n=148(Training set) No specific quantity has been explicitly stated	Single center	lncRNA XLOC_009167	Not calibrated	0.7398 (0.6493–0.8303)	78.7%	61.8%	(67)
Liang et al. (2020)	Prospective clinical trial study	China(2018.9-2022.3) No specific quantity has been explicitly stated	Multicenter	ctDNA Methylation Classifier, Physician Cancer Probability Estimates, Validated Lung Nodule Risk Models	Not calibrated	0.83	82.5%	83.3%	(83)
Xing et al. (2021)	Retrospective cohort study	China (2019.1-2020.6) n=110(Training set) n=100(Validation set)	Single center	PTGER4*, RASSF1A*, SHOX2*, nodule diameter	Not calibrated	0.943(0.891-0.995)	92.0%	96.0%	(84)
He et al. (2025)	Prospective cohort study	China (2019.1-2023.3) n=210(Training set) n=82(Validation set)	Single center	SHOX2*, SCT*, HOXA7*, age, size	Excellent	0.932 (95%Cl: 0.872–0.992)	89.1%	82.8%(	(85)
Leung et al. (2020)	Prospective study	UK (2009.1-2018.5) n=192(Training set) No independent validation set	Single center	ctDNA mutations in KRAS*, EGFR*, TP53*	Not calibrated	Not reported	75%	89%	(87)
Mathios et al. (2021)	Prospective study	American (2012–2013) n=365(Training set) n=431(Validation set)	Multicenter	cfDNA fragmentation profiles (DELFI*), CEA*	Not calibrated	0.93 (0.90–0.97)	94%	80%	(88)
Huang et al. (2020)	Retrospective case-control study	China (2014–2018) n=624(Training set) No independent validation set	Single center	7-autoantibody panel (p53, PGP9.5, SOX2, GAGE7, GBU4-5, MAGEA1, CAGE)*	Not calibrated	0.775(0.740–0.810)	50.1%	82.0%	(91)
Xu et al. (2022)	Retrospective cohort study	China (2017.08-2020.06) n=933(Training set) No independent validation set	Single center	P53*, PGP9.5*, SOX2*, GAGE7*, GBU4-5*, MAGEA18, CAGE*, age, gender, smoking history, history of previous tumors, maximum nodule diameter, number of nodules, nodule composition, vascular sign, spiculation sign, lobulation sign, pleural traction sign, cavitation sign	Not calibrated	0.96	96.4%	79.1%	(92)
Pan et al. (2020)	Retrospective cohort study	China (2015–2018) n=277(Training set) n=266(Validation set)	Single center	Final 6-marker panel:3 IgA-autoantigens (BCL7A, TRIM33, MTERF4) + 3 IgG-autoantigens (CTAG1A, DDX4, MAGEC2) *	Not calibrated	Not reported	68.2%	87.0%	(93)
Guan et al. (2023)	Retrospective cohort study	China (2018.4-2020.12) n=573(Training set) n=275(Validation set)	Single center	Serum amino acids, Orn*, C16*, C5DC*, age, gender	Excellent	0.81	74%	76%	(97)
Xu et al. (2023)	Retrospective cohort study	China (2021.3-2022.3) n=100 No specific quantity has been explicitly stated	Single center	CT*, CEA*, CYFRA21-1*, palmitic acid, W3*, nervonic acid	Excellent	Not reported	81.6%	86.8%	(98)

AUC, area unde curve; FR+-CTC, Folate Receptor-positive Circulating Tumor Cells; CEA, Carcinoembryonic Antigen. CYFRA21-1, Cytokeratin 19 Fragment 21-1; NSE, Neuron-Specific Enolase; CA125, Cancer Antigen 125. CA19-9, Cancer Antigen 19-9; PNAIDS, Pulmonary Nodules Artificial Intelligence Diagnostic System; CAC, Circulating Abnormal Cell; RMRP, RNA component of mitochondrial RNA processing endoribonuclease; NEAT1, Nuclear Enriched Abundant Transcript 1; TUG1, Taurine Upregulated Gene 1. (uAI), unbiased Artificial Intelligence; MALAT1, Metastasis Associated Lung Adenocarcinoma Transcript 1; GAS5, Growth arrest-specific 5; SOX2OT, SOX2-overlapping transcript. CTA-384D8.35. a cancer/testis antigen-associated long noncoding RNA. PGM5-AS1, PGM5 antisense RNA 1; ANRIL, antisense non-coding RNA in the Inhibitor of Cyclin-Dependent Kinase 4 locus; SFTA1P, surfactant associated 1 pseudogene; PTGER4, Prostaglandin E Receptor 4; RASSF1A, Ras Association Domain Family 1 Isoform A; SHOX2, Short Stature Homeobox; SCT, Secretin; HOXA7, Homeobox A7; ctDNA, circulating tumor DNA; KRAS, Kirsten rat sarcoma viral oncogene homolog; EGFR, epidermal growth factor receptor; TP53, tumor protein p53. anti-p53, tumor protein 53. anti-PGP9.5, protein gene product 9.5. anti-SOX2, sex-determining region Y-box 2. anti-GAGE7, G antigen 7. anti-GBU4-5, DEAD-box RNA helicase GBU4-5. anti-MAGEA1, melanoma-associated antigen A1; DELFI, DNA Evaluation of Fragments for Early Interception. anti-CAGE, cancer-associated gene protein; BCL7A, B-cell CLL/lymphoma 7A; TRIM33, Tripartite motif-containing protein 33; MTERF4, Mitochondrial transcription termination factor 4; CTAG1A, Cancer testis antigen 1A; DDX4, DEAD-box helicase 4; MAGEC2, Melanoma antigen family C2; PRDX2, Peroxiredoxin-2; PON1, Paraoxonase 1; APOC3, Apolipoprotein C3. pro-SFTPB, proprotein form of Surfactant Protein B; FGB, Fibrinogen beta chain; FGG, Fibrinogen gamma chain; Orn, Ornithine; C16, Palmitoylcarnitine; C5DC, Butyrylcarnitine; CT, Computed Tomography; W3, omega-3 polyunsaturated fatt; acids,

### 3.1 Lung cancer-related protein markers

In recent years, protein marker assays related to lung cancer have emerged as significant tools for the qualitative diagnosis of pulmonary nodules. Commonly utilized biomarkers include CEA, CYFRA21-1, NSE, and SCC. A diagnosis model built by trichotomizing CEA, CYFRA21–1 and NSE, achieved an AUC of 0.88, with a sensitivity as 0.79 and a specificity as 0.80, clearly superior to any single marker (0.63–0.78) (23). Besides, a single-center retrospective study combining CT-radiomics nomogram with CEA and CYFRA21-1, showed an AUC of 0.76 (24). Another multimodal model fusing protein markers, CT metrics and clinical features distinguished malignant from benign nodules, showing an AUC of 0.904 with 81.4% sensitivity and 90.1% specificity (25). Similarly, a study developed a four-protein plasma panel (CA125, CEA, pro-SFTPB, CYFRA21-1) integrated with clinical-imaging data that distinguished indeterminate nodules, showing an AUC of 0.95 with 62% sensitivity and 95% specificity (26). All the models-built combing with multiple classical tumor biomarkers achieved an improved diagnostic efficiency of lung cancer than single tumor biomarker did. However, the sensitivity of this model is still not satisfactory.

Beyond conventional tumor biomarkers, diverse signaling and immunomodulatory proteins driving lung cancer development are expected to sharpen the discrimination of benign versus malignant nodules. The fibrinogen beta chain (FGB) and fibrinogen gamma chain (FGG) are two subunits of fibrinogen. A preliminary study revealed that patients with malignant pulmonary nodules exhibited significantly elevated expression of FGB and FGG in plasma exosomes compared to those with benign pulmonary nodules (fold change > 1.5, p < 0.05) (27). The combination of plasma exosomal FGB and FGG were found to discriminate malignant from benign lung nodules at AUC 0.794 (81% sensitivity, 70% specificity) (28). In addition, the surfactant protein B precursor (Pro-SFTPB) was reported to be capable to predict the risk of lung cancer development (29). A proof-of-principle study from the Integrative Analysis of Lung Cancer Etiology and Risk (INTEGRAL) Consortium showed that a panel of four circulating protein biomarkers (CA125, CEA, CYFRA 21–1 and Pro-SFTPB) could be used to identify high-risk individuals for lung cancer screening, demonstrating an AUC of 0.83 (30). Although these novel protein biomarkers are promising, multiomics, *in vitro*, *in vivo* and large cohort studies are needed for the screening and validation of these biomarkers, to further improve the diagnostic efficiency.

### 3.2 Circulating tumor cell

CTCs—tumor cells shed into circulation that retain malignant morphology and molecular signatures—are captured by immunomagnetic, microfluidic or size-based enrichment and serve as liquid-biopsy biomarkers for diagnosis and treatment monitoring (31). Previous study employed an immunomagnetic bead assay to quantify folate receptor-positive CTC (FR+ CTC) and demonstrated that their efficiency in distinguishing between benign and malignant nodules with a sensitivity of 78.6% and a specificity of 68.8% (32). The FR+ CTC model in this study offers a minimally invasive method using a blood draw to detect circulating tumor cells by targeting folate receptor-alpha, specific to lung adenocarcinoma. In addition, Ren D et al. constructed an interpretable nomogram integrating pan-epithelial keratin-positive (CK7/19/panCK^+^, CD45^-^) CTC counts with three-dimensional malignant risk stratification from the unbiased Artificial Intelligence (uAI) platform CT, based on 76 surgically confirmed pulmonary nodule patients (33). Internal validation via 1,000 bootstrap iterations yielded an AUC of 0.805 (95% CI: 0.705–0.905) for the combined model, significantly outperforming either CTC count alone (AUC 0.743, 95% CI: 0.622–0.864) or uAI imaging alone (AUC 0.730, 95% CI: 0.606–0.854). Additionally, Vimentin-positive circulating tumor cells (Vim+ CTCs) (34), Vascular Endothelial Growth Factor Receptor positive circulating tumor cells (VEGFR2+ CTCs) (35), Programmed Death-Ligand 1 positive circulating tumor cells (PD-L1+ CTCs) (36) were also associated with lung cancer diagnosis.

Due to tumor cell heterogeneity, limitations in the CTC capture strategies using either biomolecular markers or size-dependence are obvious. Charge-mediated CTC isolation (CMCTCI) has been applied in cell specific targeting overcoming the above limitations (37–40). The charged nanoprobes (NPs) provide new bio-electricity-based cell targeting and capturing that is not relying on any protein-based biomarkers whose specificity has been a universal issue. Since the glycolytic-regulated surface negative charge is the hallmark characteristic of all cancer cells regardless of genotypic differences, the charge-based targeting will be unique, exclusive, and highly specific only to those that exhibit significant glycolysis. This separation technique requires no antibody labelling and is not constrained by tumor heterogeneity. Using merely 1 mL of peripheral blood, it achieves efficient, broad-spectrum capture of 2–8 circulating tumor cells within 20 minutes, while only 0–1 circulating tumor cell was detected in blood samples from 10 healthy donors (41).

As CTCs are extremely rare and heterogeneous cells in the bloodstream (a few as 0–1/7.5ml) and the majority are cleared by the immune system or perish in the circulation (42, 43), further studies are needed to improve the detection and isolation platform of CTC.

### 3.3 Circulating genetically abnormal cell

Circulating genetically abnormal cells are cells found in the bloodstream that possess acquired genetic mutations or chromosomal abnormalities (44). These cells are often indicative of serious pathological processes, most notably cancer, where they can detach from tumors and circulate (45). Their detection and analysis are crucial in oncology for early diagnosis, monitoring treatment response, and assessing the risk of metastasis (46). The detection primarily relies on analyzing blood samples to identify genetically abnormal cells or cell-free DNA (cfDNA). This is achieved through advanced techniques like polymerase chain reaction (PCR), DNA sequencing, and fluorescence *in situ* hybridization (FISH) to pinpoint specific mutations or chromosomal abnormalities (47).

CAC in conjunction with CT scans for the diagnosis of lung nodules by using the Pulmonary Nodules Artificial Intelligence Diagnostic System (PNAIDS), showed an AUC at 0.847 (48). In addition, the Lung LB™ model was built in prospective and multicenter study using CAC for the diagnosis of lung cancer. The Lung LB™ is a 4-color fluorescence *in-situ* hybridization assay for detecting circulating genetically abnormal cells (CGACs) from peripheral blood. The model demonstrated a 77% sensitivity and a 72% specificity (49). The study indicates combined CAC analysis boosts diagnostic accuracy and spares invasive biopsies.

These finding suggests that CAC can complement CT imaging, offering enhanced support for the early detection of lung cancer. This model shows promise for early diagnosis of pulmonary nodules as a non-invasive tool but needs further validation due to its limited sample size, single-center design, and lack of external validation. The complexity and cost of CAC detection may limit routine use, and standardization of these methods requires more research.

### 3.4 Circulating tumor RNA

#### 3.4.1 miRNA

MiRNAs, a category of small non-coding RNAs with gene-regulatory functions, have shown significant potential in the diagnosis of tumors (50). Circulating miRNAs are resistant to multiple freeze–thaw cycles (51). The noninvasiveness and stability make circulating miRNAs a potential tool to identify diagnostic markers in oncology. Various methods exist for miRNA extraction, including traditional Trizol reagent extraction, commercial column extraction kits, and magnetic bead enrichment. Detection techniques for miRNAs comprise reverse transcription quantitative polymerase chain reaction (RT-qPCR), digital PCR, Northern blotting, capillary gel electrophoresis, homogeneous multiplexed detection methods, and next-generation sequencing (NGS) (52).

A predictive model incorporating a plasma four-biomarker panel (miR-21-5p, miR-574-5p, CEA, CYFRA21-1) plus clinical and imaging features distinguished benign from malignant lung nodules with 80% positive predictive value (PPV), 89.5% negative predictive value (NPV) and AUC 0.921 (53). In addition, a model developed by integrating plasma miR-126, miR-210, and miR-205-5p, clinical features, and imaging characteristics exhibited excellent diagnostic efficiency for breast cancer, with an AUC 0.87, and sensitivity and specificity, achieving rates of 89.9% and 90.9% (54). Besides, research has demonstrated that a predictive model incorporating serum miR-15b-5p, miR-16-5p, and miR-20a-5p is effective in distinguishing early-stage non-small cell lung cancer (NSCLC) cases from healthy individuals (55). For testing the utility of miRNAs as a minimally-invasive diagnostic biomarker, Yu et al. investigated the expression of miR-92-a2 in the plasma of Small Cell Lung Cancer (SCLC) patients and healthy controls. The study revealed significant overexpression of plasma miR-92a-2 levels in SCLC patients compared to controls and specificity and sensitivity of 100% and 56%, respectively, along with an AUC of 0.761 was obtained for the diagnosis of SCLC (56).

Moreover, a panel comprising 34 miRNAs has demonstrated the capability to identify individuals with early-stage NSCLC among asymptomatic high-risk populations, achieving an accuracy rate of up to 80% (57). This miRNA-based diagnostic test, which utilizes a signature of 13 specific miRNAs, was administered to over 1,000 high-risk participants in the Continuous Observation of Smoking Subjects (COSMOS) study (58). The test exhibited an overall diagnostic accuracy for lung cancer of 75% (95% CI 72–78) (59).Additionally, the other studies have found that circulating miR-25 (60), miR-233 (60), miR-21 (61) were upregulated in lung cancer, while miR-486-5p (62), miR-126 (61), miR-30d (61), miR-30d (61), miR-30e-5p (61) and miR-451 (61) were downregulated, indicating potential application in the diagnosis of lung cancer.

However, the inconsistency in miRNA evaluation makes this method less applicable in clinical settings. A standard control, standardized isolation method and large cohort are needed to improve the reliability of the results.

#### 3.4.2 LncRNA

Long non-coding RNA (lncRNA) represents a category of non-coding RNA molecules exceeding 200 nucleotides in length, which are intricately associated with tumorigenesis and tumor progression. In sufficient quantity, tumor-derived lncRNA typically form a highly stable secondary structure, which is resistant to ribonuclease activity and is thus stable in peripheral blood, making lncRNA suitable for quantitative detection (63).

A diagnostic model incorporating four-lncRNAs panel (RNA component of mitochondrial RNA processing endoribonuclease [RMRP], Taurine up-regulated gene 1 [TUG1], Nuclear paraspeckle assembly transcript 1 [NEAT1] and Metastasis associated lung adenocarcinoma transcript 1 [MALAT1]) achieved AUCs of 0.89, outperforming traditional lung cancer-related tumor marker combinations (CEA, CA125, and CYFRA21-1) for the diagnosis of adenocarcinoma (64). LncRNA Sex-Determining Region Y Box 2 overlapping transcript (SOX2OT), antisense noncoding RNA in the antisense non-coding RNA in the Inhibitor of Cyclin-Dependent Kinase 4 locus (ANRIL), combined with traditional tumor biomarkers CEA, SCCA, and CYFRA21–1 were selected to form a diagnostic panel for NSCLC (65). Higher specificity and sensitivity were observed in the panel both in test and validation set compared to single biomarkers. Furthermore, plasma lncRNAs Growth Arrest-Specific 5 (GAS5) and SOX2OT combined as dual-gene diagnostic model distinguished NSCLC from benign nodules with an AUC of 0.82 (77%–87%), with 83.8% sensitivity and 81.4% specificity (66). To distinguish between lung cancer and pneumonia, lncRNA eXpressed LOCus 009167 (XLOC_009167) was found to be elevated in the whole blood of lung cancer patients and achieved an AUC of 0.7005 and a sensitivity of 90.1% (67). In addition, the single lncRNAs including LncRNA 152, GAS5, Cytoskeleton regulator RNA (CYTOR), membrane-associated guanylate kinase inverted 2 antisense RNA 3 (MAG12-AS3) and zinc finger antisense 1 (ZNAS1) have been shown to hold potential to serve as a diagnostic marker distinguishing NSCLC from benign lung disease (63, 68).

### 3.5 Circulating tumor DNA

Tumor-released ctDNA, genetically mirroring its tissue origin, offers a non-invasive biomarker for early detection, treatment monitoring, and prognosis of cancer (69). Cell death, active secretion through Extracellular Vesicles (EVs) and lipoprotein complexes, CTC disruption, chromosomal instability, and external factors like anti-tumor therapies, all contribute to the increased release of ctDNA (70, 71). Current methodologies for extracting ctDNA from blood samples include magnetic bead-based, in silico column-based, and liquid-phase extraction techniques. Several studies have demonstrated the potential of ctDNA (Tumor Protein p53 [TP53], Retinoblastoma 1 [RB1]) as a valuable tool for the diagnosis and prognosis of SCLC (72, 73). Oncogenic driver gene alterations within ctDNA are typically identified using single-gene PCR and NGS technologies (74). ctDNA can be classified into various types according to its characteristics, commonly including methylated ctDNA, mutated ctDNA, and copy number variant ctDNA (75).

Tumor-derived ctDNA carries cytosine phosphodiester bond guanine-island (CpG-island) 5-methylcytosine (5-mC), a chemical modification that can be detected in blood (76). Hypermethylation of the CpG islands in promoter regions of tumor-suppressor genes has been shown to contribute to carcinogenesis (77). Several studies have reported the potential of investigating tumor-specific methylations in blood for screening and diagnosis of lung cancer. For example, various gene promoters were found to be differentially methylated in ctDNA between patients with lung cancer and controls, including short stature homeobox 2 (SHOX2) (78, 79), doublecortin like kinase 1 (DCLK1) (80), septin9 (SEPT9) (81), ras association domain family 1 isoform A (RASSF1A), and retinoic acid receptor B2 (RARB2) (82).

In a multicenter observational trial of 10,560 patients with 0.5–3 cm non-calcified nodules, a ctDNA methylation model distinguished benign from malignant disease with 82.5% sensitivity and 83.3% specificity (83). Similarly, fusing ctDNA methylation (Prostaglandin E Receptor 4 [PTGER4]/RASSF1A/SHOX2) with imaging lifted lung-nodule classification to AUC 0.951, outperforming image-only or traditional Mayo-type models and validating the “epigenetics-plus-imaging” paradigm (84). In addition, A SHOX2/Secretin (SCT)/Homeobox A7 (HOXA7) ctDNA methylation panel plus clinical variables yielded AUC 0.87, reinforcing the robustness of methylation-based models for benign–malignant nodule discrimination (85). Prior work centered on ctDNA methylation, while mutation-bearing ctDNA fragments are likewise being explored for early cancer detection (86). In a study analyzing mutant ctDNA in blood of patients, it was found to exhibit good diagnostic performance as a biomarker for lung cancer diagnosis (87). It showed high specificity (89%), sensitivity (75%) and PPV (98%), underscoring its utility for flagging malignancy. Moreover, DNA Evaluation of Fragments for Early Interception (DELFI), a machine-learning tool that profiles ctDNA fragmentation, distinguished lung cancer from non-cancer in 781 symptomatic individuals (AUC = 0.90); adding clinical variables and CEA lifted the AUC to 0.93 (88).

### 3.6 Tumor-associated autoantibodies

Tumor-associated autoantibodies are host immunoglobulins directed against aberrantly expressed or mutation-generated antigens (89). These autoantibodies enable early detection of lung cancer, guide therapy monitoring, and support prognostic assessment (90).

A four-autoantibody panel (tumor protein 53 [p53]/Sex-Determining Region Y-Box Transcription Factor 2 [SOX2]/G antigen 7 [GAGE7]/Glycosylated β-subunit 4–5 [GBU4-5]) detected by enzyme-linked immunosorbent assay (ELISA) distinguished early-stage lung cancer from benign nodules with AUC 0.764, outperforming single-marker tests and establishing a multiplexed model that balances sensitivity (0.478) and specificity (0.814) (91). Similarly, integrating seven-autoantibody (P53, protein gene product 9.5 [PGP9.5], SOX2, GAGE7, GUB4-5, melanoma-associated antigen A 1 [MAGEA1], and cancer antigen gene [CAGE]) signatures with clinical and imaging data raised early-stage lung-nodule diagnosis to 59.7% sensitivity and 81.1% specificity, lifting the ROC-AUC from 0.748 to 0.96 (92). Another study that adds plasma IgA to IgG against TIF1γ raised the AUC from single-IgG levels to 0.734, capturing mucosal immunity and yielding clinically useful early-lung-cancer detection (93). The Early Cancer Detection Test-Lung (EarlyCDT-Lung) test a prospective autoantibody panel for lung-cancer detection, yielded 9–16% six-month PPV in 1613 high-risk patients, depending on six-autoantibody (p53, New York Esophageal Squamous Cell Carcinoma 1 [NY-ESO-1], CAGE, GBU4-5, SOX2, Human Neuronal Protein D [HuD]) versus seven-autoantibody (p53, NY-ESO-1, CAGE, GBU4-5, SOX2, HuD, Melanoma-associated antigen 4 [MAGE A4]) model (94).

In summary, these findings underscore the potential of integrating autoantibody signatures with clinical-imaging data enables accurate, non-invasive early diagnosis of lung cancer.

### 3.7 Lung cancer-related metabolites

During the development of tumors, metabolites in the body change. More recently, researchers have turned to metabolomics to analyze specific metabolic markers for the early diagnosis of lung cancer (95). Metabolomics offers a novel perspective in this context and can provide real-time reflections of cellular status (96).

A plasma metabolomics–eXtreme Gradient Boosting (XGBoost) model built from 478 cancers and 370 benign nodules distilled 16 features (demographics, six amino acids, eight acyl-carnitines) to yield 0.81 AUC, 74% sensitivity and 75% specificity, pinpointing ornithine and palmitoyl-carnitine as non-invasive early markers and validating metabolomics-guided risk stratification of pulmonary nodules (97). Additionally, integration of chest CT features (location, lobulation, spiculation, vascular convergence), elevated serum CEA/CYFRA21-1, and a plasma fatty-acid signature (palmitate, stearate, docosahexaenoic acid [DHA], α-linolenic acid [ALA], etc.) accurately discriminated 72 malignant pulmonary nodule (MPN) from 38 benign pulmonary nodule (BPN), underscoring the added value of multi-modal imaging–metabolite–marker synergy for non-invasive nodule characterization (98). A clinical study of 65 non-smoking female NSCLC patients, 6 benign lung-tumor cases and 65 healthy controls identified the cysteine/serine/1-monooleoylglycerol panel as a biomarker signature for diagnosing non-smoking female NSCLC (99).

Moreover, numerous studies have identified metabolites associated with lung cancer. Gas Chromatography–Mass Spectrometry (GC-MS) plasma profiling by Musharraf et al. showed lung-cancer patients had elevated fatty acids and glucose versus Chronic Obstructive Pulmonary Disease (COPD), healthy smoker and non-smoker controls (100). Besides, Ding et al. linked lung cancer to dysregulated glucose metabolism, citing elevated glycerol-3-phosphate, lactate, acetyl-CoA and 3-phosphoglycerate (101). Lung-cancer sera display increased glycerophospholipids and hypoxanthine alongside divergent gut-microbiome and metabolome profiles relative to healthy subjects (102). In addition, a seven-metabolite microbiota-derived panel (uracil [Ura], histamine [His], cysteine [Cys], 3-hydroxypicolinic acid [HPA], uric acid [UA], indoleacrylic acid [IA], and fatty acid [FA]) reliably distinguishes early-stage lung adenocarcinoma from healthy controls (103).

Ultimately, metabolomics-anchored, multidimensional models merging metabolic, imaging and clinical data promise accurate, non-invasive triage of pulmonary nodules for early lung-cancer care. However, Low metabolite identification accuracy hampers discovery of effective lung-cancer biomarkers.

## 4 Artificial intelligence based predictive models

Against the backdrop of numerous data points, AI excels at extracting features from large-scale lung-nodule imaging data and building robust predictive models that help clinicians rapidly and accurately distinguish benign from malignant nodules, markedly improving diagnostic precision and efficiency (104). AI-based lung-nodule predictors fall into two streams: classical machine-learning models that rely on hand-crafted features (Logistic Regression [LR], Linear Discriminant Analysis [LDA], Support Vector Machine [SVM], Decision Tree [DT], Random Forest [RF], Gradient Boosting Tree [GBT], K-Nearest Neighbors [KNN], etc.) and deep-learning models (CNN, DBN, etc.) that autonomously learn hierarchical patterns via multi-layer neural nets (105).

In recent years, the integration of AI tools with medical imaging has enabled a new generation of models to detect cancer, representing a significant leap in precision diagnostics. For example, an AI-radiomics logistic model integrating age and CT features (–350 Hounsfield Unit [HU] Consolidation-to-Tumor Ratio [CTR] ≥ 50%) showed stable malignancy prediction for subsolid nodules, achieving AUCs of 0.721 (training) and 0.757 (validation) in 370 nodules (106). This study confirmed that AI-radiomics model delivers individualized, non-invasive malignancy risk estimates for central pulmonary nodules. Similarly, a study implemented Grid-tuned hyper-parameter optimization lifted SVM to 99.2% accuracy on a small Kaggle lung-cancer set, outperforming optimized XGBoost, DT and LR, yet larger multi-disease and prospective data remain essential for clinical translation (107).

As manually engineered features plateaued, the field naturally turned to deep learning, where convolutional networks automatically discover hierarchical imaging patterns and now dominate pulmonary-nodule classification. A deep-learning Lung Cancer Prediction-Convolutional Neural Network (LCP-CNN) model outperformed the Brock model (AUC 0.896 vs 0.868), offering a non-invasive, accurate alternative for pulmonary nodule malignancy prediction (108). Going deeper, an M-ResNet combining residual blocks and pyramid pooling captured multi-scale CT features of complex lung nodules on Lung Image Database Consortium and Image Database Resource Initiative (LIDC-IDRI), delivering AUC 0.928 (95% CI: 0.917–0.938) to classify benign and malignant pulmonary nodules, and setting a robust baseline for nodule characterization (109). Besides, a prospective 260-nodule Zhongshan Hospital cohort (2018–2021) demonstrated that the AI-assisted diagnostic platform σ-Discover/Lung V1.0.2—an integrated deep-learning framework that probabilistically classifies pulmonary nodule benignity or malignancy from clinical CT imaging—achieved 75.8% accuracy, 89.6% sensitivity, 48.3% specificity and AUC 0.755, corroborating its practical utility in routine lung-nodule management (110). Moreover, novel dense architectures D1/D2 attained 99.96% mean 10-fold accuracy on colon/lung histopathology and CT (Lung and Colon Cancer Histopathological Image Dataset [LC25000], National Clinical Trial-Colorectal Cancer [NCT-CRC], etc.), outperforming ResNet50, Xception and seven other baselines with only 10% of the data, offering a robust, multimodal boost to cancer imaging (111). These studies presented the key characteristics of the AI-based predictive models for lung cancer (**Table 3**).

**Table 3:** key characteristics of AI-based diagnostic models for lung cancer.

Authors (year)	Study design	Population	Data source	Predictors used	Calibration	AUC* (95%Cl)	Sensitivity	Specificity	Reference
Shi et al. (2025)	Retrospective cohort study	China (2018.01-2023.12) n=259(Training set) n=111(Validation set)	Multicenter	Age, solid component volume, nodule mean CT value	Excellent	0.757(0.632-0.881)	82.0%	82.6%	(106)
Syed et al. (2024)	Retrospective cohort study	No specific quantity has been explicitly stated	Single center	Gender, Age, Wheezing, Swallowing difficulty, yellow fingers, Chronic Disease, Anxiety, Coughing, Alcohol. Chest pain, Allergy, Smoking, Peer pressure, Shortness of breath, Fatigue	Not calibrated	0.992	100%	Not reported	(107)
Baldwin et al. (2020)	Retrospective cohort study	UK (2018.01-2019.08) No reported training set n=1397(Validation set)	Multicenter	LCP-CNN AI Model: Extracts solely CT nodule image features (deep learning) *	Not calibrated	0.896(87.6-91.5)	99.57%	28.0%	(108)
Batool et al. (2025)	Retrospective cohort study	Pakistan No specific quantity has been explicitly stated	Single center	Bone X-ray images	Not calibrated	0.928(0.917–0.938)	99.98%	99.95%	(109)
Zhang et al. (2023)	Retrospective cohort study	China (2018.1-2021.4) n=260 No specific quantity has been explicitly stated	Single center	CT imaging characteristics (density, position, size, etc.)	Not calibrated	0.755	89.6%	48.3%	(110)
Uddin et al. (2024)	Retrospective cohort study	No specific quantity has been explicitly stated	Multicenter	Multimodal images	Not calibrated	0.99	99.96%	99.99%	(111)

AUC, area under curve; LCP-CNN, Lung Cancer Prediction Convolutional Neural Network.

In addition, other emerging AI techniques also facilitate disease diagnosis which are promising in the application of lung nodules prediction. For example, Multi-Strategy Parrot Optimizer (MSOP) refines breast-cancer imaging. Deep learning sharpens forensic bone-age assessment (112). Besides, Inception-V4 paired with Dynamic Snow Leopard boosts diabetic-retinopathy grading (113). As the development of conversational AI in healthcare, ChatGPT helps children with Down syndrome enhance emotional recognition (114).

To conclude, AI-based predictive models for lung cancer have evolved decisively from classical algorithms to advanced deep learning, establishing themselves as powerful tools for precision diagnostics. Their successful application in prospective studies underscores a tangible path toward broader clinical implementation, heralding a new era in oncological care. Looking forward, the critical next steps involve transcending pure performance metrics to address the challenges of real-world integration——including model interpretability, multi-center data standardization, and seamless workflow adoption—to fully realize the promise of AI in routine pulmonary nodule management and beyond.

## 5 Multimodal predictive model

Recent advances in AI, especially deep learning, have significantly improved the differentiation of pulmonary nodules in medical imaging. Studies show that clinical phenotypes (like age and smoking history) and biomarkers (such as serum tumor markers and gene mutations) are linked to whether nodules are benign or malignant. By combining clinical, imaging, and biomarker data with AI through multimodal approaches, more accurate models for predicting the malignancy risk of pulmonary nodules can be developed.

A study created a multimodal deep learning model (clinical-biomarker-combined deep radiomics [CB-DR]) combining clinical data, CT radiomics, and biomarkers TPI-1 and miR-206, validated externally (115). The model showed excellent diagnostic performance with an AUC of 0.90, 90% accuracy, 90% sensitivity, and 82% specificity. Besides, by adding adipose-tissue radiomics that mirror the tumor micro-environment, the deep-learning radiomics clinical nomogram (DLRCN) lifted CT discrimination of 6–30 mm nodules to AUCs ≥ 0.946 in both internal and external cohorts, outperforming clinical-only (AUC 0.80), intranodular-plus-perinodular (0.80), fat-only (0.86), Mayo (0.56) and Brock (0.59) models, while Decision Curve Analysis (DCA) and significant Net Reclassification Improvement (NRI)/Integrated Discrimination Improvement (IDI) gains (p < 0.05) confirmed the added value of fat-derived imaging biomarkers (116). Moreover, in a study Fusing CT morphology, AI probability, serum Vascular Endothelial Growth Factor (VEGF) and a 7-autoantibody panel for 176 histology-proven nodules, a logistic model reached AUC 0.946 (training) and 0.856 (external validation, 80% sensitivity, 86% specificity), outperforming imaging-only, AI-only and any single-biomarker approach, with significant NRI/IDI (p < 0.05) and superior DCA net benefit, underscoring the value of multimodal biomarker integration for early lung-cancer diagnosis (117). It summarizes the key characteristics of the multimodal predictive models that have been applied to lung cancer, including their study design, sample size, predictors and reported performance metrics (**Table 4**).

**Table 4:** key characteristics of multimodal diagnostic models for lung cancer.

Authors (year)	Study design	Population	Data source	Predictors used	Calibration	AUC* (95%Cl)	Sensitivity	Specificity	Reference
Wei et al. (2025)	Prospective study	China (2021–2023) n=176Training set) n=51(Validation set)	Multicenter	Clinical characteristics (gender, smoking history), imaging characteristics (size, location, lobulation), biomarkers (TPI-1, miR-206), deep learning-based radiomics features*	Excellent	0.90(0.81–0.97)	90%	82%	(115)
Miao et al. (2024)	Retrospective cohort study	China (2016–2022) n=158(Training set) n=390(Validation set)	Multicenter	Deep learning features (extracted by BiVGG), adipose tissue radiomics features, clinical characteristics (age, gender, BMI, smoking history, etc.) *	Excellent	I-T: 0.946(0.936–0.955);E-T1: 0.948(0.933–0.963);E-T2: 0.962(0.945–0.979)	I-T: 85.3%;E-T1: 87.5%;E-T2: 87.1%	I-T: 88.2%;E-T1: 85.7%;E-T2: 90.7%	(116)
Zhou et al. (2025)	Retrospective cohort study	China n=454(Training set) n=117(Validation set)	Single center	AI extraction: lobulation sign, bronchial inflation sign, AI-predicted malignancy probability, part-solid nodule nature, nodule diameter, solid component proportion, VEGF*, TP53*, PGP9.5*, SOX2*, GAGE7*, GAGE4-5*, MAGEA1*, CAGE*	Excellent	0.856	80%	86%	(117)

AUC, area under curve; BiVGG, Bilateral VGG. BMI, Body Mass Index. TPI-1, Triosephosphate isomerase-1. VEGF, Vascular Endothelial Growth Factor. TP53, Tumor Protein p53. PGP9.5, Protein Gene Product 9.5. SOX2, SRY-box Transcription Factor 2. GAGE7, G Antigen 7. GAGE4-5, G Antigen 4–5. MAGEA1, Melanoma Antigen Family A, 1. CAGE, Cancer Antigen Gene.

In summary, multimodal predictive models that incorporate clinical data, imaging characteristics, biomarkers, and artificial intelligence have the potential to improve the accuracy of differentiating between benign and malignant pulmonary nodules. Future research should prioritize the ongoing optimization of algorithmic performance and the validation of its stability and generalizability across diverse populations. This will support the broader integration of this technology into clinical practice.

## 6 Discussion

The rapid evolution of predictive models for lung nodule malignancy reflects a paradigm shift from relying on single-modal data to integrating multimodal approaches (118). While classical clinical-imaging models like the Mayo (13), VA (15), and Brock (16) models laid the foundational groundwork, they exhibit limitations in generalizability across diverse populations and ethnicities, as seen in the suboptimal performance of the Mayo model in Chinese cohorts. This underscores the critical need for extensive, multi-center, and cross-population validation to ensure that predictive tools are robust and equitable. Models developed from specific demographics, such as the VA model’s focus on elderly male veterans, risk significant performance decay when applied to the general population, including women and younger individuals. Future model development must prioritize prospective, multinational cohorts that capture global demographic and genetic diversity to build truly generalizable and clinically reliable tools.

There are a few studies validated their models across-populations. For example, Artificial Neural Network (ANN) and support vector machine with least absolute shrinkage and selection operator (SVM-LASSO) models were trained on 113 Italian Continuous Observation of Smoking Subjects (COSMOS) nodules. External validation on 72 the United States-Image Database Resource Initiative (US-LIDC) nodules dropped AUC only modestly (ANN 0.89→0.82; SVM-LASSO 0.90→0.86), and both still significantly outperformed Lung-RADS (0.76), with Delong/McNemar tests indicating stable cross-center performance (119). In addition, a separate investigation employed the XGBoost-based PKU-M model, trained on 1,739 Chinese MPNs (AUC 0.91, Brier 0.122), retained 0.89 across six Chinese plus one Korean center (n=583) and 0.87 prospectively (n = 200), outperforming Brock, Mayo, VA and clinicians while boosting sensitivity +14% and specificity +8% (120). The aforementioned studies demonstrate that radiomics models retain discriminative power across cohorts, yet true multi-ethnic, multicenter trials remain scarce. Future work must couple multi-site validation with ensemble learning to reconcile population-specific biomarker heterogeneity and harden model robustness.

The exploration of novel biomarkers, including CTCs, CACs, ctDNA (especially methylation patterns), and various RNA species, holds immense promise for enhancing diagnostic precision (121). However, the journey from discovery to clinical application is fraught with challenges. Key among these is the development of standardized, cost-effective, and highly specific detection methods. For instance, while charge-based CTC isolation offers a promising solution to tumor heterogeneity, and NGS unlocks the potential of ctDNA methylation signatures, these technologies require further refinement and simplification for routine clinical use. The inconsistency in miRNA evaluation methodologies also highlights a broader issue in the biomarker field: a lack of standardized protocols for sample processing, analysis, and validation (122). The future of biomarker discovery likely lies in high-throughput multi-omics approaches, but this necessitates large-scale, collaborative studies to identify and validate signatures with high specificity and clinical utility.

**Figure 1:**
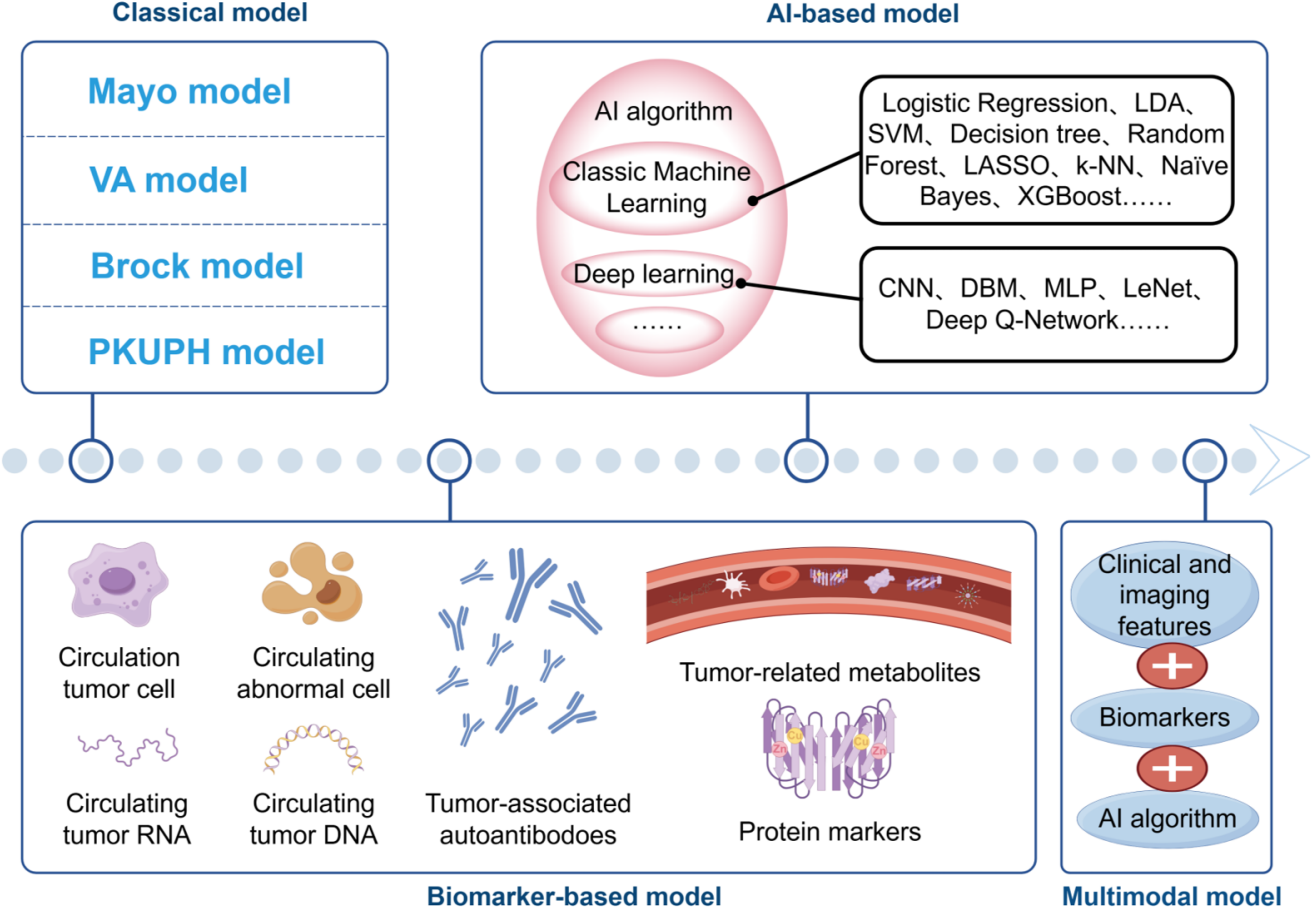
The evolving landscape of predictive models. Classical clinical-imaging models (Mayo, VA, Brock and PKUPH) rely on logistic regression of demographic and CT variables. Biomarker-based approaches incorporate CTCs, CACs, circulating tumor DNA/RNA, proteins, autoantibodies and metabolites, either alone or in combination with clinical features. AI-based models progress from conventional machine learning algorithms (SVM, Random Forest, XGBoost, etc.) to deep-learning networks (CNN, DBN, MLP, etc.). Contemporary multimodal integration fuses clinical, imaging, biomarker and AI algorithms, offering a non-invasive pipeline for personalized early lung cancer detection.

AI, particularly deep learning, has revolutionized the analysis of complex imaging data, often surpassing human interpretation and traditional models like Brock (123). AI’s ability to autonomously extract subtle radiographic features has significantly improved nodule characterization. Nevertheless, its application raises two significant concerns: data dependency and security. AI models are often hampered by limited generalizability when trained on non-diverse, single-source datasets, leading to biases (124). Furthermore, the use of large-scale, multi-institutional datasets for training necessitates robust data security frameworks, including federated learning, to protect patient privacy while enabling collaborative model improvement. A concise diagram illustrating the classification of various models (**Figure 1**).

Data security is a cornerstone in the development of big data and AI-based diagnostic models. robust security measures are not merely a regulatory compliance issue but a fundamental ethical and clinical necessity for building reliable and trustworthy AI tools in medicine. To meet data security requirements for 3D models, a dual-layer encryption and steganography mechanism utilizing memristive coupled neural networks has been developed (125). This mechanism establishes a highly secure data protection system, resistant to brute-force attacks, statistical analysis, and linear and differential attacks. It accomplishes this through hyperchaotic key generation, dual-layer encryption, an ultra-large key space, NIST randomness verification, secure S-box design, and a fusion mechanism for encryption and steganography. The presented algorithm can reliably ensure privacy for sensitive digital data transmissions and storage applications. Future work should focus on integrating such hardware-efficient security layers directly into AI diagnostic pipelines to ensure end-to-end privacy from data acquisition to clinical decision-making.

Looking forward, the most promising application lies in multimodal models that seamlessly integrate clinical parameters, AI-enhanced imaging, and liquid biopsy biomarkers. Studies combining CT radiomics with adipose-tissue features, serum autoantibodies, or protein panels have consistently demonstrated superior performance (AUCs >0.90) compared to any single-modality approach. The primary challenge for clinical translation is the transition of these sophisticated models from research settings to routine practice. This requires the development of user-friendly interfaces, validation in real-world clinical workflows, and clear evidence of cost-effectiveness. Ultimately, the future of lung nodule management will be guided by these integrated, data-driven tools, enabling more personalized and precise patient care, reducing unnecessary invasive procedures, and improving early detection rates of lung cancer.
